# Establishing a follow-up of the Swiss MONICA participants (1984-1993): record linkage with census and mortality data

**DOI:** 10.1186/1471-2458-10-562

**Published:** 2010-09-21

**Authors:** Matthias Bopp, Julia Braun, David Faeh, Felix Gutzwiller

**Affiliations:** 1 Institute of Social and Preventive Medicine (ISPM), University of Zurich, Hirschengraben 84, 8001 Zurich, Switzerland

## Abstract

**Background:**

To assess the feasibility and quality of an anonymous linkage of 1) MONICA (MONItoring of trends and determinants in CArdiovscular disease, three waves between 1984 and 1993) data with 2) census and mortality records of the Swiss National Cohort in order to establish a mortality follow-up until 2008. Many countries feature the defect of lacking general population cohorts because they have missed to provide for follow-up information of health surveys.

**Methods:**

Record linkage procedures were used in a multi-step approach. Kaplan-Meier curves from our data were contrasted with the survival probabilities expected from life tables for the general population, age-standardized mortality rates from our data with those derived from official cross-sectional mortality data. Cox regression models were fit to investigate the influence of covariates on survival.

**Results:**

97.8% of the eligible 10,160 participants (25-74y at baseline) could be linked to a census (1990: 9,737; 2000: 8,749), mortality (1,526, 1984-2008) and/or emigration record (320, 1990-2008). Linkage success did not differ by any key study characteristic. Results of survival analyses were robust to linkage step or certainty of a correct link. Loss to follow-up between 1990 and 2000 amounted to 4.7%. MONICA participants had lower mortality than the general population, but similar mortality patterns, (e.g. variation by educational level, marital status or region).

**Conclusions:**

Using anonymized census and death records allowed an almost complete mortality follow-up of MONICA study participants of up to 25 years. Lower mortality compared to the general population was in line with a presumable ‚healthy participant' selection in the original MONICA study. Apart from that, the derived data set reproduced known mortality patterns and showed only negligible potential for selection bias introduced by the linkage process. Anonymous record linkage was feasible and provided robust results. It can thus provide valuable information, when no cohort study is available.

## Background

Surveys that assess clinical and lifestyle properties have been conducted in many countries. However, only few of these surveys provide a mortality follow-up. This substantially limits their potential to evaluate the significance of risk factors in the population. An exception are countries with national health data registers [1] or with an established system for ascertaining vital status, e.g. the National Death Index in the U.S. [2], the Canadian record linkage system [3] or the Western Australia data linkage system [4]. However, all these record linkage systems use names or a unique personal identification number (PIN). In most countries, for confidentiality reasons, names and PINs are not available to researchers. The combination of anonymized population register or census data with a mortality register may offer a resort. In Switzerland, the Swiss National Cohort (SNC), a nationwide anonymized record linkage of census and mortality records, meets these requirements [5]. With respect to person years and number of deaths, the SNC is one of the largest longitudinal datasets worldwide. Thanks to its design, the SNC allows the linkage of data from health surveys and clinical studies conducted in the past. This promises a mortality follow-up of study participants in an elegant and efficient manner.

The MONICA (MONItoring of trends and determinants in CArdiovscular disease) study is an international multicentre project initiated and coordinated by the World Health Organization [6]. In Switzerland, three waves of the MONICA study have been conducted between 1983 and 1992 [7,8]. Unfortunately, the opportunity to provide for follow-up information was missed and thus no survival analyses could be performed. MONICA participants appear to be ideal candidates for linkage with the SNC because of the large number of participants, the vast amount of variables and the proximity in time to electronically processed censuses. With follow-up periods between 17 and 25 years, a sufficient number of deaths can be expected to warrant sufficient robustness of analyses.

With this study, we aimed at 1) establishing and testing a procedure allowing to link MONICA data with the censuses 1990/2000, emigration and mortality records 1984-2008; 2) evaluating potential for selection bias due to differences in linkage success between subgroups; 3) analyzing and comparing survival probabilities in population groups with different socio-demographic properties. Approval for the linkage of MONICA and SNC data was obtained from the Ethics Committee of the Canton of Zurich.

## Methods

### MONICA data

We included all three population studies from Switzerland conducted within the framework of MONICA: MONICA I (1984-86, N = 3,445), MONICA II (1988-89, N = 3,466), MONICA III (1992-93, N = 3,252, table 1). The identical two-stage sampling procedure consisted of 1) drawing a sample of 51 out of 651 communities after stratification according to their size and 2) drawing the subjects from the population files of the communities (resident population aged 25-74 years in Vaud/Fribourg and aged 35-64 in Ticino; for details see [7]). The selected persons were invited to attend a health examination in their community of residence and to complete a self-administered questionnaire. Weight and height were measured, blood pressure, blood lipids and other biological parameters were obtained following standardized procedures methods of the MONICA Project described previously [7-9].

**Table 1:** Characteristics of MONICA participants by study wave and by canton, Switzerland 1984-1993

	Study wave			Study region (canton)		
		
	MONICA I	MONICA II	MONICA III	Vaud*	Fribourg*	Ticino
Participants (n)	3,442	3,466	3,252	4,263	1,378	4,519
						
Age of inclusion (years)	25-74	25-74	25-74	25-74	25-74	35-64
Women (%)	49.1	48.6	51.4	49.9	48.8	49.8
Foreign nationals (%)	14.7	18.6	21.2	17.7	7.4	21.8
Age (in years, mean)	47.6	47.8	47.8	47.0	45.7	49.0
						
University education (%)	6.5	7.7	8.5	9.0	6.5	6.5
Tertiary education (%)	14.0	10.2	11.6	16.5	12.8	7.4
Upper secondary education (%)	44.7	50.4	50.6	48.3	45.1	49.8
Mandatory education (%)	34.8	31.8	29.3	26.1	35.6	36.4
						
Never married (%)	13.4	12.0	12.1	13.6	15.8	10.5
Married (%)	76.8	76.9	76.0	73.7	77.4	79.0
Widowed (%)	4.2	4.1	3.7	4.2	3.6	4.0
Divorced (%)	5.6	6.9	8.2	8.5	3.3	6.5
						
Start of examination VD/FR**	29.10.1984	02.11.1988	23.11.1992			
Duration VD/FR (days)	222	240	215			
Start of examination TI**	11.11.1985	24.10.1988	28.10.1992			
Duration TI (days)	198	185	210			

*together belonging to the predominantly French speaking region "Vaud/Fribourg"

**equal to study entry

VD/FR: Vaud/Fribourg area; TI: Ticino area

MONICA: MONItoring of trends and determinants in CArdiovscular disease

In total 15,893 individuals were sampled. Of these, 348 (2.2%) had to be excluded because they had died or moved out. From the remaining 15,545 eligible persons 1,388 (8.9%) could not be contacted, 3,422 (22.0%) refused and 572 (3.7%) didn't follow the appointment, leaving 10'163 (65.4%) individuals which could be examined. Respective participation rates for the three study waves (MONICA I, II, III) were 60, 63 and 54% in Vaud/Fribourg, and 78, 74 and 76% in Ticino (see also [9]). Overall, 10,160 participants aged 25-74 at baseline were available for record linkage (3 participants of MONICA I were excluded because of incomplete date of birth).

### Swiss National Cohort data

The SNC encompasses all residents of Switzerland enumerated in the national 1990 or 2000 census. Deterministic and probabilistic methods of record linkage were used to link anonymized census records to death or emigration records [5]. Of the 6.874 million individuals registered in the census of 12/04/1990, 6.9% could not be linked to either a census 2000 record, a death record 1990-2000 or an emigration record 1990-2000. The majority of unlinked records concerned individuals aged 10-29 years [5], which only marginally concerned the MONICA study population in the Vaud/Fribourg region. Swiss census enumeration and registration of deaths occurring in Switzerland (including cause of death information) are virtually complete. Registration of deaths - but not necessarily of cause of death - of Swiss nationals abroad should be fairly complete. However, for foreign nationals residing in Switzerland, registration of deaths occurring abroad is incomplete.

In order to adopt "best practices" to avoid the possibility to identify individuals [10], the SNC is not a permanent single database including all available datasets but a temporary link of relevant datasets. Linkage with additional datasets needs a special data contract with the Swiss Federal Statistical Office. Only the link tables produced for the projects are archived. For every project a customized analysis file is produced that only contains the previously defined SNC variables.

### Linkage of MONICA and SNC

In order to determine vital status of MONICA participants, we used record linkage procedures including all potential identification variables, i.e. variables available in MONICA and in the census. Minimal required information for a promising record linkage was sex, exact date of birth and place of residence. Additional helpful identification variables were nationality, marital status, educational category and profession.

Swiss censuses assess not only the current place of residence but also the community of residence five years before the census (i.e. 1985 in 1990 census and 1995 in 2000 census). This information helped to retrieve MONICA participants who moved between sampling and census. Deaths which occurred before the 1990 census were not covered by the standard SNC and had to be evaluated separately for potential linkage. Therefore, study participants not retraceable in the SNC, i.e. in the 1990 census, had to be evaluated separately for a potential link with the official death registry. For deaths occurred before the 1990 census, linkage success is expected to be slightly lower.

Since also small communities were included in two or even three MONICA waves, the same individual could possibly be sampled more than once. As a preliminary step, all participants with identical sex/date of birth/community were checked for repeated sampling, with a plausibility test based on profession, body height and weight and blood pressure.

Record linkage between MONICA and SNC was performed step by step, with satisfactorily linked individuals excluded from succeeding steps (MONICA III, which was conducted in 1992/3, is not involved in the steps 2 and 5, because there are no deaths occurred before 1990 among participants):

1) MONICA community = 1990 census community of residence

2) Participants of MONICA I, II: MONICA community = community in mortality records (only deaths occurred before 1990 census)

3) MONICA community = 1985 community of residence (based on 1990 census)

4) MONICA community = 2000 or 1995 community of residence (based on 2000 census)

5) Participants of MONICA I, II: linkage with other community based on mortality statistics (only deaths occurred before the 1990 census)

6) linkage with other community of 1990 census in the same canton

7) linkage with community of 1990 census in another canton

8) manual control and optimizing (check of remaining unlinked MONICA participants for potential partner records in the 1990 census and the 1984-90 death records; typically these records showed discordances regarding date of birth, place of residence and occupation which had prevented automated record linkage, but considering all available information and potential alternative links strongly suggested that the records referred to the same individual)

MONICA wave, region of residence (Vaud/Fribourg, Ticino), age, sex, nationality (Swiss or foreign), marital status and educational level (mandatory, upper secondary, tertiary, university education) were included as independent variables in a logistic regression model to analyse the odds for linkage failures or loss to follow-up between the censuses 1990 and 2000.

### Survival analysis and test for heterogeneity

Survival time was defined as the time between study entry (i.e. date of examination), and either 1) date of death (from mortality records) or 2) the last potential date of death (12/31/2008), which serves as censoring time point. Persons who were found in the 1990 census but neither in the 2000 census nor in a mortality record 1991-2000, were censored on 12/04/1990, and emigrants on emigration date 1991-2008. Individuals who could neither be found in the 1990 or 2000 census nor in the mortality records were excluded.

Kaplan-Meier curves were used to visualize survival probabilities; they decrease when a death occurs, while a censored observation is marked by a tick-mark, leaving the curve unchanged. For the estimation of hazard ratios, a Cox regression model was fit including relevant independent variables (age, sex, marital status, educational level, nationality) and adjusting for the subgroups study region and study wave. Multivariable Cox regression was also used for sensitivity analysis (control for heterogeneity and errors introduced in the linkage process).

Additionally, survival curves were contrasted with the survival probabilities expected in the general Swiss population, using national life tables of the Swiss Federal Statistical Office [11]. Life tables were only available for periods around the census, with 1998-2003 as the most recent one. Because yearly information is required for the calculation of expected survival, we used the respective rates as reference values for the year in the middle of the interval (e.g. the year 2000 for the last interval). Rates for the years between the reference values were obtained using weighted means. For example, the rate for 1992 was obtained by using the weighted mean of the rate from the 1990 period with weight eight and from the 2000 period with weight two. For the calculation of rates for the years 2001-2008, we conducted extrapolations of progress of life expectancy from preceding years.

Each person in the linked data set was matched to a fictitious person from the life tables according to sex, age and year of study entry. Therewith, the expected survival probability of each individual could be calculated and combined to expected population survival probabilities [12,13]. This can be plotted and compared with the estimated survival probabilities of the group of interest. A one-sample logrank test was used to compare expected and observed survival probabilities [14,15]. Also, based on age-specific and age-standardized death rates from our data, visual and descriptive comparisons to official cross-sectional death rates can be drawn.

General descriptive analyses and survival estimations were performed with Stata 11 (Stata Corp, Texas, USA), the calculation of expected survival was performed in R version 2.9.2 (The R Foundation for Statistical Computing, 2009).

## Results

### Record linkage

Generally, even in larger communities, the combination of sex, date of birth and community was very specific. In most cases, accepted links were corroborated by concordant occupational and/or educational information. Seventy-two individuals (of whom 59 from the canton of Ticino) appear to have been examined twice: 33 in MONICA I and II, 16 in MONICA I and III, 23 in MONICA II and III. Relying on body height, weight and blood pressure, we concluded in three cases that two MONICA participants with identical sex/date of birth/community were different individuals.

**Figure 1:**
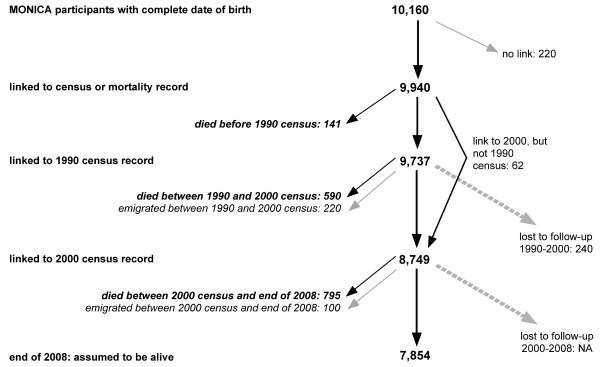
**MONICA Switzerland 1984-93 and Swiss National Cohort (SNC) until 2008: chart of linked participants**. MONICA: MONItoring of trends and determinants in CArdiovscular disease. NA: not available, can only be determined when 2010 census will be linked.

For 9,940 out of 10,160 eligible MONICA participants (97.8%) a link to a census or mortality record could be established (figure 1). 87 of these concerned MONICA III participants who could be linked to the preceding (1990) census, but not to a subsequent record (2000 census, death or emigration), leaving 9,853 individuals for survival analysis.

For 9,737 participants a link to the 1990 census was found and for 8,749 a link to the 2000 census (8,687 to both, 1990 and 2000 censuses). Overall 1,526 individuals could be linked to a death record and 320 to an emigration record.

240 persons could be linked to the 1990 but neither the 2000 census nor a succeeding death or emigration record. Including 220 matches with emigration records, loss to follow-up between 1990 and 2000 amounted to 4.7%. Since there was no census at the end of the study, loss to follow-up after the 2000 census could not be determined, i.e. all 7,854 individuals linked to the 2000 census but not to a succeeding death or emigration record are assumed to have survived.

By far the most linkage matches were obtained with community of residence (i.e., identical in MONICA and 1990 census, 8,721 or 89% of all census links). Additional matches for 838 individuals could be established relying on community of residence in 1985, 1995 or 2000 (8.6% of all census links). Matches involving other communities of residence than those indicated in MONICA added 196 census records. Finally, 44 matches with census records could be found by manual search. From the 141 matches with a death occurred before the 1990 census, 109 relied on MONICA community of residence, 30 on other communities of residence and two on manual search.

Difference in linkage success according to any of the key study characteristics - and therefore potential for selection bias - was marginal (Additional file 1: Table S1). Logistic regression models used to compare not linked (N = 220) with linked MONICA participants and those lost to follow-up between the censuses in 1990 and 2000 (but not emigrated, N = 240) with those who could be followed up to the 2000 census or a mortality record between 1990 and 2000 showed no significant differences (not shown).

### Kaplan-Meier plots

**Figure 2:**
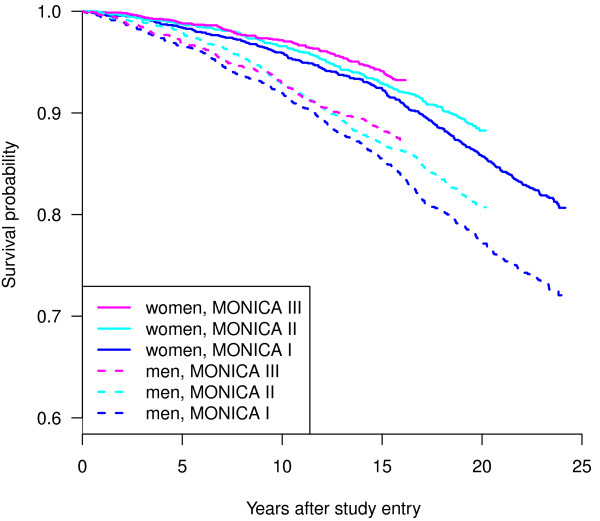
**Survival of participants in the three MONICA waves in Switzerland 1984-1993 linked to the Swiss National Cohort: Kaplan-Meier curves by wave and sex (N = 9,940)**. MONICA: MONItoring of trends and determinants in CArdiovscular disease.

Figure 2 shows a Kaplan-Meier plot of the three MONICA waves, separately for men and women. Because of the later study entry, the respective follow-up times for participants of MONICA II and III are shorter. Generally, men and women belonging to the same MONICA wave show similar patterns (however, on different levels).

**Figure 3:**
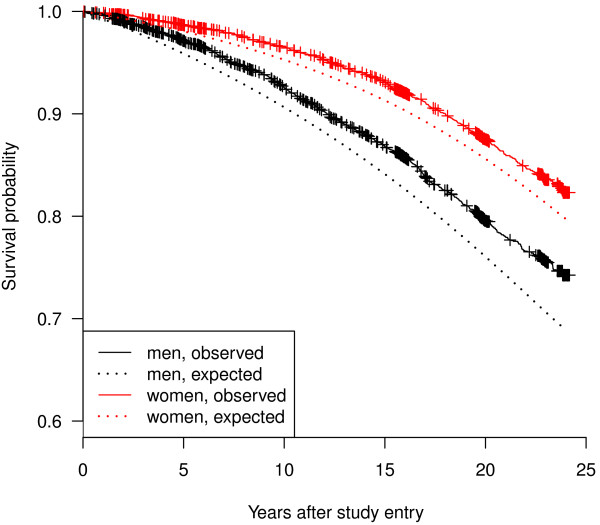
**Survival of participants in the three MONICA studies in Switzerland 1984-1993 compared to the Swiss general population: Kaplan-Meier curves by sex (N = 9,940)**. MONICA: MONItoring of trends and determinants in CArdiovscular disease.

Figure 3 displays by sex expected survival curves obtained from official Swiss life tables and Kaplan-Meier curves obtained from our data set. In both cases, the expected survival curves are below the Kaplan-Meier curves with slightly increasing distance. There are striking aggregations of censoring indicators in both Kaplan-Meier curves. These are explained by the distribution of study entry dates. If all participants had entered each wave on the same date, only one censoring indicator per MONICA wave would be visible. In contrast, the indicators would be about equally distributed over the study period if MONICA had no waves and the participants had entered the study at random time points. In fact, participants of each MONICA wave entered the study at different dates within a time interval of few months (see table 1), causing the strongly clustered censoring points in the right part of the curves. The study entry intervals of MONICA I in Ticino and Vaud/Fribourg differed (table 1), which explains the two small clusters close to each other. The similar clusters to the left of the plot relate to persons from MONICA I and II which could only be followed up to the 1990 census. In accordance with the curves, the one-sample log-rank test showed significant differences (p <0.00001) for both sexes. These differences applied to both regions (data not shown).

### Cox regression

Hazard ratios for survival resulting from the Cox regression model including confidence intervals and p-values are given in table 2. A hazard ratio significantly below one means higher survival probability. Inclusion of age, sex, MONICA wave and region (Vaud/Fribourg, Ticino) lead to significant differences in survival. Being married tended to be associated with higher survival, but higher hazard ratios were only significant for divorced persons. Educational level showed a consistent gradient, with progressively increasing survival with better education. In contrast, nationality had no influence on mortality. Sensitivity analyses using Cox models did not show any noteworthy difference of survival in function of linkage step or certainty of a correct link, when sex, age and wave was adjusted for.

**Table 2:** Cox regression derived adjusted* hazard ratios for survival 1984-2008, 9,817 participants of the Swiss MONICA study with educational and marital status information (accumulating 1,519 deaths)

Variable	Hazard ratio	95% Confidence interval	p-value
Age at study entry (years)	1.107	(1.101-1.113)	< 0.001
			
MONICA I (1984/85)	1		
MONICA II (1988/89)	0.802	(0.712-0.904)	< 0.001
MONICA III (1992/93)	0.777	(0.671-0.900)	0.001
			
Vaud/Fribourg region	1		
Ticino region	0.868	(0.777-0.969)	0.012
			
Women	1		
Men	2.126	(1.904-2.373)	< 0.001
			
University education	1		
Tertiary education	1.322	(0.992-1.762)	0.057
Upper secondary education	1.499	(1.173-1.915)	0.001
Mandatory education	1.700	(1.328-2.176)	< 0.001
			
Married	1		
Never married	1.157	(0.962-1.391)	0.121
Widowed	1.069	(0.881-1.299)	0.498
Divorced	1.257	(1.018-1.552)	0.033
			
Swiss nationals	1		
Foreign nationals	0.993	(0.848-1.163)	0.927

MONICA: MONItoring of trends and determinants in CArdiovscular disease

*adjusted for all covariates

### Age-standardized rates

Because there are regional mortality differences, the national life table used for the Kaplan-Meier-curves are not ideal. In order to demonstrate this, a table that compares the study populations with the general population of the VD/FR region is included. Due to small numbers and the more limited age range at baseline in Ticino, a comparison with official mortality statistics only makes sense for the Vaud/Fribourg region since 1985. In the 1980 s, mortality rates of MONICA participants were clearly lower than in the general population (table 3). In the 1990 s this difference disappeared in men and substantially decreased in women. Since 2000, male MONICA participants appear to be again at lower mortality risk than the general population, whereas female participants approximated the general population.

**Table 3:** Comparison of age-standardized mortality rates (per 100,000 person years, WHO standard population "Europe", ages 45-79) between study and general population of the Vaud/Fribourg region

	Linked data set MONICA/SNC (1)	Data of Swiss Federal Statistical Office (2)	Ratio (1)/(2), %
Males 1985-89	1305.8	1880.5	69.4
Males 1990-94	1432.4	1721.8	83.2
Males 1995-99	1530.4	1546.8	98.9
Males 2000-04	1097.5	1345.7	81.6
Males 2005-08	723.3	1185.6	61.0
N deaths	386	47,491	
			
Females 1985-89	512.4	867.4	59.1
Females 1990-94	606.6	810.2	74.9
Females 1995-99	587.9	737.4	79.7
Females 2000-04	525.6	672.8	78.1
Females 2005-08	552.1	611.6	90.3
N deaths	198	28,928	

MONICA: MONItoring of trends and determinants in CArdiovscular disease

## Discussion

We evaluated whether it was possible to link census and mortality records with data from population studies conducted up to 25 years ago without having available names or a unique personal identification number. Our anonymous record linkage proved to be an elegant and cost-effective way to establish a mortality follow-up of clinical and behavioural studies which otherwise could not have been further exploited. Only 2.2% of the Swiss MONICA study participants 1984-1993 could not be traced in the census or the mortality records.

We could not determine loss to follow-up for the entire observation time (1984-2008) but only between the 1990 and 2000 census. Nevertheless, the 4.7% (220 emigrants plus 240 individuals which could not be traced at all) lost to follow-up found in our study can be considered as low. The first National Health and Nutrition Examination Survey (NHANES I) hat a loss to follow-up of 5.6% between 1971-75 and 1982-84 [16]. However, the NHANES I had a much more elaborated, extensive and costly design. Even from a 25 year perspective, the presumptive loss to follow-up can be expected to be well below the critical threshold of 20% stipulated for cohort studies [17,18].

We used several standard procedures in order to evaluate the quality and usability of the linked data set and to look for potential selection bias. Most of the observed variations in mortality were in the expected direction. This applies especially for the differences by age and sex, but also for marital status and educational level. The significantly higher survival in Ticino than in Vaud/Fribourg is in line with cross-sectional studies [19,20]. The general progress in life expectancy in the last decades became evident when comparing survival between MONICA I and III.

The clearly lower mortality rates of MONICA participants compared to the general population in the 1980 s are in line with other health survey studies. In an Austrian cohort with voluntary medical examination, Klenk et al. observed an even larger difference, with a mortality rate in participants nearly 40% lower than in the general population [21]. However, in general, in cohorts, this difference is expected to be highest at study entry. Thereafter participants are expected to approximate the general population, because in the long run risks of most chronic diseases become similar. Therefore this „healthy participants advantage" is likely to decrease in succeeding years/decades. However, such an effect has only been described for migrants [22]. Nevertheless, the increasingly lower mortality rates in male participants compared to the male general population after 2000 are unexpected. The difference is too large to be substantially explained by loss to follow-up between the 2000 census and the end of 2008. The larger difference in men than in women could relate to a lower MONICA participation rate in men. This could have led to a stronger "healthy participant" selection in men than in women.

This study has several limitations. If the rate is unrelated to exposure status, false-positives (linked records not belonging to the same person) and false-negatives (unlinked records belonging to the same person) attenuate risk differences toward the null and, hence, dilute any true effect [3]. However, false-positives attenuate also risk ratios toward the null and false-negatives lead to a loss of power. It's therefore worth to look for potential misclassification.

Variations in loss to follow-up were small and thus unlikely to result in differential misclassification. However, since there was no census at the end of the study period (2008) and thus only incomplete determination of loss to follow up between 2000 and 2008, differential misclassification cannot completely be ruled out. Some potential for bias may also arise from non-participation, a problem more often observed in Vaud/Fribourg than in Ticino. Those who agree to participate in a study are more healthy than those who refuse [23,24]. This is likely to explain why overall mortality figures for our study participants were lower than those of the general population.

Persons lost to follow-up after 2000 had to be assumed to have survived. Since they contribute person time but no deaths, their mortality risk is underestimated. However, those expected to have higher loss to follow-up (e.g. divorced and lower educated individuals) still had significantly increased hazard ratios. Thus patterns of relative mortality risks are unlikely to be substantially biased. Finally, our procedure only allows the linkage with cause-specific mortality data but not with morbidity data. However, since hospital discharge data is available in Switzerland, there is potential for such a linkage.

## Conclusions

When instead of individual names or unique personal identification numbers there is only a kind of population registry, establishing a mortality follow-up of cross-sectional studies is nevertheless feasible, even decades after enrolment. In our example, proportions of unlinked and lost to follow-up individuals were negligible and socio-demographic characteristics didn't substantially differ from successfully linked and followed individuals. Comparison of survival didn't show significant differences by nationality, linkage step or certainty of a correct link. Potential for differential misclassification should thus be very small. Also, most variations in survival between participants and the general population were explainable.

The investment in the record linkage necessary for this task and the complexity of the methods are modest. Its results may in many respects be comparable to those that could be expected from a much more extensive cohort study that only provides results after a long latency. Our approach is thus of particular interest for resources-constrained settings and for countries without ongoing general population cohort studies. A wealth of clinical and behavioural information can be reawakened and enhanced with specific sociodemographic information from the censuses. This opens the door to entirely new possibilities and therewith substantially generates added value to surveys and retrospective cohorts for which informed consent is not generally available. Finally, our method also allows to assess the representativeness of population studies in terms of survival. Most public health interventions address entire populations. However, they are often based on health surveys with limited representativeness. Better understanding of this discrepancy could uncover hidden potential for public health intervention.

## Competing interests

The authors declare that they have no competing interests.

## Authors' contributions

MB conceived the study, conducted the record linkage, sketched a first draft and repeatedly read and corrected later versions. JB assisted in the record linkage, conducted survival analyses and tests for heterogeneity and wrote part of the manuscript. DF calculated crosstables, wrote the respective text, added background knowledge and substantially improved the manuscript by repeated readings and rephrasing as well as critical discussions of the intellectual content. FG contributed important intellectual content and gave final approval of the version to be published. All authors read and approved the final manuscript.

## Pre-publication history

The pre-publication history for this paper can be accessed here:

http://www.biomedcentral.com/1471-2458/10/562/prepub

## Supplementary Material

Additional file 1 **Appendix**. Table S1- Proportion of linked MONICA participants, Switzerland 1984-1993. Click here for file
