# Supplementary material for: Establishing a follow-up of the Swiss MONICA participants (1984-1993): record linkage with census and mortality data
Source: BMC Public Health. 2010 Sep 21;10:562. doi: 10.1186/1471-2458-10-562 (PMC2955001; doi:10.1186/1471-2458-10-562)
Supplement: Additional file 1 — Appendix. Table S1- Proportion of linked MONICA participants, Switzerland 1984-1993. [file 1471-2458-10-562-S1.DOC]

**Appendix**

**Table S**1 - Proportion of linked MONICA participants, Switzerland 1984-1993

|  |  |
| --- | --- |
| Variable | % |
| All participants | 97.8 |
| MONICA I (1984-86) | 96.6 |
| MONICA II (1988-89) | 98.2 |
| MONICA III (1992-93) | 98.8 |
| Vaud/Fribourg region | 97.8 |
| Ticino region | 97.9 |
| Women | 97.5 |
| Men | 98.1 |
| Swiss nationals | 97.8 |
| Foreign nationals | 97.8 |
| Age 25-39 years | 97.2 |
| Age 40-54 years | 98.1 |
| Age 55-74 years | 98.1 |
| University education | 97.6 |
| Tertiary education | 97.2 |
| Upper secondary education | 98.2 |
| Mandatory education | 97.6 |
| Never married | 96.8 |
| Married | 98.2 |
| Widowed | 97.8 |
| Divorced | 95.7 |
|  |  |

MONICA: MONItoring of trends and determinants in CArdiovscular disease
